# Discovery of a *Katablepharis* sp. in the Columbia River estuary that is abundant during the spring and bears a unique large ribosomal subunit sequence element

**DOI:** 10.1002/mbo3.206

**Published:** 2014-08-28

**Authors:** Peter Kahn, Lydie Herfort, Tawnya D Peterson, Peter Zuber

**Affiliations:** 1 Center for Coastal Margin Observation & Prediction and Institute of Environmental Health, Oregon Health & Science University3181 SW Sam Jackson Park Road, Mail code HRC3, Portland, Oregon, 97239

**Keywords:** Columbia River coastal margin, heterotrophic protist diversity, *Katablepharis* CRE, unique sequence element

## Abstract

Heterotrophic protists play significant roles in pelagic food webs as bacterivorous and herbivorous consumers. However, heterotrophic protists—unlike autotrophic ones—are often difficult to track since they tend to lack features such as photosynthetic pigments that allow for remote sensing or for bulk characterization. Difficulty in the identification of heterotrophic protists has often resulted in lumping them into broad groups, but there is a strong need to develop methods that increase the spatial and temporal resolution of observations applied to particular organisms in order to discover the drivers of population structure and ecological function. In surveys of small subunit rRNA, gene (SSU) sequences of microbial eukaryotes from the Columbia River to the Pacific Ocean, the heterotrophic flagellate *Katablepharis* sp. were found to dominate protist assemblages (including autotrophic and heterotrophic fractions) in the spring, prior to the freshet. We discovered a 332 base pair unique sequence element (USE) insertion in the large subunit rRNA gene (28S) that is not present in other katablepharids or in any other eukaryote. Using this USE, we were able to detect *Katablepharis* within mixed assemblages in river, estuarine, and oceanic samples and determine spatial and temporal patterns in absolute abundance through quantitative PCR and fluorescence in situ hybridization. Given their high abundance and repeatable temporal patterns of occurrence, we hypothesize that the Columbia River Estuary *Katablepharis* (*Katablepharis* CRE) plays an important role in estuarine biogeochemical and ecosystem function.

## Introduction

Heterotrophic protists play significant roles in pelagic food webs as bacterivorous and herbivorous consumers (Pomeroy 1974; Azam 1983), as food sources for organisms at higher trophic levels such as metazoans (Gifford 1991), and as remineralizers of essential nutrients such as nitrogen and phosphorus (Caron et al. 1990). Heterotrophic protists, particularly small cells (<20 *μ*m), are often difficult to assign taxonomically using light or electron microscopy, as many of them lack distinctive morphological features or characteristic pigments. As a result, they have often been placed into broad groups, such as “heterotrophic nanoflagellates” (2–20 *μ*m), which could include a wide range of organisms of different taxonomic groups bearing different metabolic potentials and that play varied roles in aquatic food webs. The collection of established cultures of heterotrophic protists is also not likely to be representative of the dominant cells in the environment, as organisms that are easily cultured are often found at low abundance in natural assemblages (Lim et al. 1999). Since the advent of culture-independent molecular-based techniques, numerous studies (Diez et al. 2001; Lopez-Garcia et al. 2001; Moon-van der Staay et al. 2001; Stoeck et al. 2003; Bass and Cavalier-Smith 2004) have begun to report unexpectedly high levels of diversity within the autotrophic, mixotrophic, and heterotrophic protist populations in diverse aquatic environments. For example, these studies have uncovered several novel lineages within the bacterivorous marine stramenopiles that can comprise a large proportion of protist populations and can be responsible for up to 10% of bacterivory and nutrient remineralization in the upper ocean (Massana et al. 2006).

The Columbia River coastal margin, which includes freshwater, brackish, and saline environments along a river-to-ocean continuum, is an ideal place to investigate the diversity of heterotrophic protists because of the broad range of environmental conditions that exist over a relatively narrow geographical region. The Columbia River coastal margin provides habitats for a wide variety of ecologically and economically important species, such as salmonids and various types of shellfish (Roegner et al. 2011). The Columbia River estuary is characterized by large amounts of allochthonous detritus from the adjacent river and ocean that is the primary source of organic matter driving ecosystem processes, with allochthonous organic matter supporting up to 84% of secondary production by the estuarine microbial populations (Simenstad et al. 1990). Yet, the overwhelming majority of research conducted on protist assemblages in the Columbia River (including the saline and freshwater reaches of the estuary) thus far has been focused on the autotrophic fraction, which is dominated by freshwater diatoms (Haertel et al. 1969; Frey et al. 1984; Lara-Lara et al. 1990; Small et al. 1990; Sullivan et al. 2001).

Previous studies on heterotrophs in the Columbia River estuary have shown that the mesozooplankton (0.2–2 mm) are composed primarily of freshwater, oligohaline, and polyhaline forms (Haertel et al. 1969; Simenstad et al. 1990), while particle-attached bacteria accounted for up to 90% of heterotrophic bacterial activity and are an important part of the estuarine food web (Crump et al. 1998). One study (Crump and Baross 1996) does suggest that nanoflagellates and oligotrich ciliates are the most common form of heterotrophic protist in the estuarine turbidity maximum, but that study was limited in spatial and temporal extent. Relatively little is known about the composition and ecological role of heterotrophic protists in the river, estuary, and plume environments, despite their likely importance in organic matter transformations within aquatic food webs that link microbial activity and higher trophic levels (Sherr and Sherr 1994, Arndt et al. 2000). Given the large amounts of particulate organic matter in the system, heterotrophic nanoplankton could play an important role in the fate of particulate organic matter in the Columbia River estuary. This critical gap in knowledge prevents the identification of trophic linkages within the aquatic food web, which could be used to inform management decisions. Higher spatial and temporal resolution of monitoring for taxa of interest, as well as improved estimates of protist diversity, are necessary in order to determine the drivers of population structure as well as biogeochemical and ecosystem function.

A molecular approach can offer valuable new insights into protist assemblage structure and diversity in the Columbia River coastal margin, particularly for the heterotrophs. To evaluate heterotrophic protist assemblages, small subunit (SSU) rRNA gene clone libraries were analyzed for water samples collected at salinities of ∼0 and ∼15 in the Columbia River estuary, and salinities of ∼28–31 in the plume in April and August 2007 and in April, July, and September 2008. Further analysis of the large subunit (LSU) rRNA gene sequences was conducted for the highly dominant heterotrophic flagellate in the mid-salinity water SSU rRNA gene clone libraries in both April 2007 and 2008, a unique katablepharid henceforth referred to as *Katablepharis* CRE (Columbia River Estuary). This analysis uncovered a 332 base pair unique sequence element (USE) within the D2 region of the LSU that shows no significant similarity to any LSU sequences in the National Center for Biotechnology Information (NCBI) database and displays an elevated GC content compared to its associated SSU and LSU rRNA sequences (data retrieved on 10 January 2014). The presence and diversity of this element were further examined to answer the following research questions:

1. What is the spatial and temporal distribution of organisms bearing this unique element amongst *Katablepharis* CRE and other katablepharids in the Columbia River coastal margin?
2. Is this unique element found in any other organisms in the Columbia River coastal margin and/or elsewhere?
3. Can the unique element be used as a taxonomic marker to facilitate ecological studies of *Katablepharis* CRE?

## Methods

### Sample acquisition

**Figure 1:**
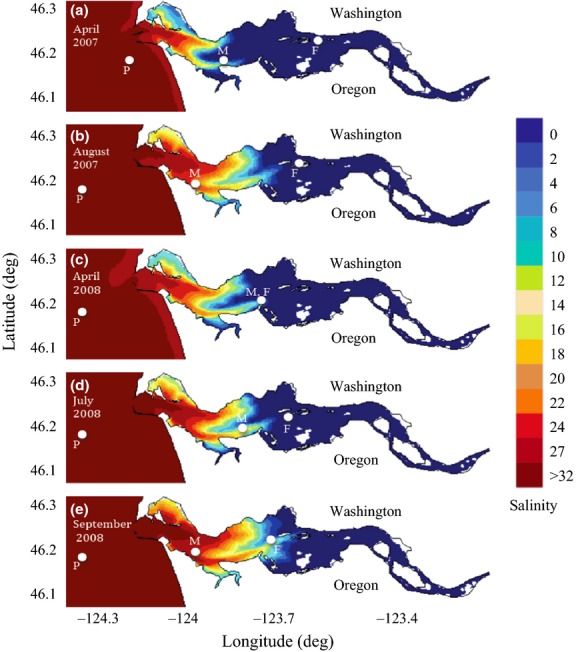
Sampling locations in the Columbia River estuary and plume for water collected in A. April and B. August 2007, as well as C. April, D. July, and E. September 2008. Samples were taken in freshwater (F; salinity of 0), mid-salinity water (M; salinity of 15), and the plume (P; salinity of 28–31). In April 2008, freshwater and mid-salinity samples were taken at the same location but at different depths (2 and 17 m, respectively). Color gradient indicates maximum bottom salinity intrusion simulations taken from the DB14 river-to-shelf simulation database (http://www.stccmop.org/datamart/virtualcolumbiariver/simulationdatabases). Note the increase in salinity intrusion (more saline water reaching further upstream) from April to August in 2007, and from April to September in 2008. Sampling sites of mid-salinity water from August 2007 and September 2008 were not located further upstream than those for April 2007 and 2008 samples, however, because August and September samples were taken towards the end of ebb tides while April samples were obtained at the end of flood tides.

**Figure 2:**
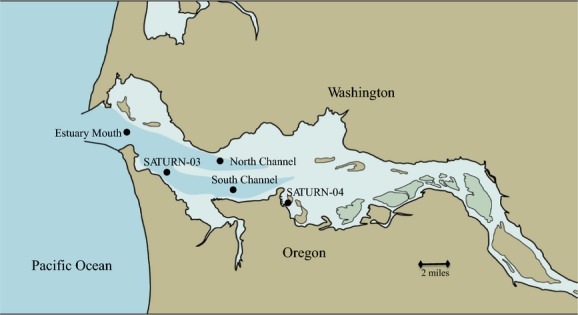
Sampling locations for qPCR and FISH analyses conducted for April-July 2013. Surface and bottom samples were taken monthly for five sites: SATURN-04, South Channel, SATURN-03, Estuary Mouth, and the North Channel.

Samples for SSU sequence analysis were collected in the Columbia River coastal margin along the river-to-ocean gradient from sites with three distinct salinities in April 2007 and 2008. Figure1 and Table1 provide the details of location, salinity, temperature, and depth for all samples used for SSU sequence analysis. Water was collected from the Columbia River estuary and its plume during April and August 2007, as well as April, July, and September 2008 aboard several vessels (M/V *Forerunner* [estuary April 2007], R/V *Barnes* [estuary August 2007] and R/V *Wecoma* [all other samples]). The Columbia River estuary consists of both a tidal brackish water region (from river and ocean water mixing) and a freshwater tidal region that extends further upstream. Freshwater and mid-salinity water samples were collected within the Columbia River estuary and were defined as having salinity values of 0 and 15, respectively. Plume water was collected outside the Columbia River bar and was defined as having a salinity of 28–31 (Barnes et al. 1972). In addition, samples for quantitative PCR and fluorescence in situ hybridization (FISH) were collected once a month from April to June 2013 aboard the M/V *Forerunner* in surface and bottom waters throughout the estuary at five sites: near the SATURN-04 observatory station (Baptista et al. 2008) in the south shipping channel of the estuary, near the SATURN-03 observatory station, in the estuary mouth, and in the north channel of the estuary (Fig.2). Surface samples were also collected near SATURN-03, south channel, and the north channel in July 2013. Figure2 and Table2 provide the details of location, salinity, temperature, depth, and turbidity for all samples used for qPCR analysis. Water was collected either from Niskin bottles attached to a Seabird 911plus CTD (conductivity- temperature-depth) rosette or with a high volume-low pressure centrifugal pump used to collect water using a PVC hose lowered alongside a Seabird 911plus CTD system.

**Table 1:** Physical characteristics of water samples collected for this study.

	Freshwater					Mid-salinity					Plume				
Apr-07	Aug-07	Apr-08	Jul-08	Sep-08	Apr-07	Aug-07	Apr-08	Jul-08	Sep-08	Apr-07	Aug-07	Apr-08	Jul-08	Sep-08
Salinity (PSU)	0.3	0.1	0.2	0.1	0.3	14.2	14.0	14.4	14.7	13.6	28.0	283	30.0	29.4	31.2
Temperature (°C)	8.9	20.6	8.9	19.6	18.5	9.7	17.9	8.9	14.5	14.8	10.1	15.1	8.8	10.8	11.3
Depth (m)	2.0	2.0	2.0	2.0	2.0	10.0	12.0	17.0	2.0	2.0	2.0	2.0	2.0	2.0	2.0

Sampling locations are given in Figure1.

**Table 2:** Salinity, temperature, and depth for samples used in qPCR analysis.

		4 April 2013			23 May 2013			20 June 2013		
	Salinity (PSU)	Temperature (°C)	Depth (m)	Salinity (PSU)	Temperature (°C)	Depth (m)	Salinity (PSU)	Temperature (°C)	Depth (m)
North channel	S	1.0	9.8	0.8	2.9	13.3	1.0	4.3	17.4	1.3
	B	26.2	9.4	20.1	28.2	9.8	23.9	26.5	14.9	20.2
Estuary mouth	S	5.8	11.0	0.8	9.9	12.4	1.1	19.1	15.7	1.2
	B	18.9	9.8	8.4	30.1	9.3	8.1	30.2	13.8	15.2
SATURN 03	S	3.5	10.1	0.8	3.3	13.2	1.0	2.1	17.8	1.0
	B	25.7	9.5	15.2	28.6	9.9	15.0	26.9	14.8	15.2
South channel	S	2.2	10.0	0.8	0.1	13.8	1.0	0.1	18.0	1.0
	B	24.2	9.5	16.4	0.1	13.8	10.2	0.4	17.9	11.2
SATURN 04	S	0.2	10.1	1.0	0.1	13.8	1.0	0.1	17.9	0.8
	B	0.7	10.0	7.1	0.1	13.8	7.3	0.0	17.9	8.4

S, refers to surface sample; B, refers to bottom sample.

### Nucleic acid extraction

After collection, water was immediately filtered through 0.2-*μ*m-pore-size Sterivex filters (polyethersulfone membrane, Eastar housing, EMD Millipore, Darmstadt, Germany) using a peristaltic pump set to low speed until it became clogged (1–5 L). One Sterivex filter was collected per sample taken. We chose not to pre-filter as we did not want to omit particle-attached unicellular microorganisms. Water was manually forced gently out of each filter using an air-filled syringe and 2 mL of the fixative RNAlater (Ambion, Life Technologies, Grand Island, NY, USA) was added to the Sterivex before freezing at −80°C aboard the ship. DNA was extracted from the particulate material from each sample using a phenol-based extraction as described in Herfort et al. (2011). Extraction was performed twice for each Sterivex filter and the two total extracts were pooled.

### PCR conditions

SSU rRNA gene DNA sequences were amplified using the eukaryote-specific primers EukA (5′-AACCTGGTTGATCCTGCCAGT-3′; position ∼1–20) and EukB (5′-TGATCCTTCTGCAGGTTCACCTAC-3′; position ∼1780–1800) (Diez et al. 2001) in a Bio-Rad, (Hercules, CA, USA) DYAD PCR thermocycler. The PCR mixture, reaction, and cleanup followed Herfort et al. (2011). PCR products were stored at −20°C.

### Cloning and sequencing

The purified PCR products were ligated with a topoisomerase 1 (TOPO) vector (pCR 2.1, Life Technologies, Grand Island, NY, USA) and the resulting constructs were then introduced by transformation into One Shot Top 10 chemically competent *Escherichia coli* cells from the TA cloning kit (Life Technologies), plated, and inoculated into two 2 mL 96-deep well plates (Thermo-Fisher Matrix, Hudson, NH, USA) following Herfort et al. (2011). Plates were stored at −80°C until sent to the Genome Sequencing Center at Washington University in St. Louis to be sequenced using the BigDye® Terminator protocol (Life Technologies) with the EukA/EukB primer set, as well as an internal primer, 528f (5′-CCGCGGTATTCCAGCTC-3′) (Elwood et al. 1985). Two 96-well plates were sequenced per sample for a total of 192 raw sequences per sample.

### SSU sequence analysis

For each SSU clone, contigs (continuous consensus sequences) were assembled using Geneious v5.3 software (Drummond et al. 2010) from the sequencing reads of the forward primer (EukA: position ∼1–20), reverse primer (EukB: position ∼1780–1800) and an internal primer (528f: position ∼528–548) to generate a ∼1800 bp sequence. Poor sequence reads (Phred score <20) and vector sequences were excluded from further analysis. Contig sequences were then searched against the NCBI nonredundant nucleotide database for homologous sequences, and those sequences that had at least 1000 bp aligned, an expectation value ≤1e-80 and percent identity ≥97% were used for further analysis. Sequences resembling multicellular organisms (for example, copepods) were removed from the data set, while those most closely related to known protist sequences were grouped according to their metabolism (autotrophic or heterotrophic), class, and genus. An average of 30 sequences per sample related to heterotrophic protist taxa was used for further analysis, and an average of 14 metazoan and 27 autotrophic sequences per sample were removed from the data set, respectively. A replicate 500 bp data set constructed with sequencing reads from the forward primer (EukA: position ∼1–20) was also used for class-level analysis (average of 42 heterotrophic sequences per sample), and confirms trends seen with the 1800 bp data set (Fig. S1). All SSU sequences have been deposited to NCBI with accession numbers KJ925152-KJ926058.

### LSU sequence analysis of *Katablepharis* CRE

A full sequence of the internal transcribed spacer (ITS) 1, 5.8S, and ITS 2 region and a partial sequence of the LSU of the rRNA gene were obtained from *Katablepharis* CRE (accession number KJ925151). A katablepharid-specific SSU forward primer (Kj3F: 5′-TGGATCGAAAGGTCTGGGTA-3′, position:1451–1471) was designed and tested for specificity against the NCBI nonredundant nucleotide database. The Kj3F primer was used with a general eukaryotic LSU reverse primer (LR9: 5′- AGAGCACTGGGCAGAAA-3′, position 2188–2204) available from the Vilgalys laboratory web site (http://www.biology.duke.edu/fungi/mycolab/primers.html). This sequencing revealed a 332 bp USE detected in the LSU region of the rRNA gene of *Katablepharis* CRE.

A suite of primers was designed to examine the presence and diversity of the USE detected in the LSU region of the rRNA gene of *Katablepharis* CRE. These PCR primers were tested for specificity against the NCBI nonredundant nucleotide database and are detailed in Table3 and in the Results section. For all PCR reactions, PCR reaction mixtures contained 1X PCR buffer, 1.5 mmol/L MgCl_2_, 0.2 mmol/L each of dATP, dCTP, dGTP, and dTTP, 0.2 *μ*mol/L of each primer, 2 units per reaction of Platinum *Taq* polymerase (Life Technologies), and 1 *μ*L template DNA (∼100 ng) in a final 25 *μ*L volume. The following PCR steps were performed: Initial denaturation at 94°C for 2 min, 30 cycles of 30 sec of denaturation at 94°C, 30 sec of annealing at 55°C, and 1 min of extension at 72°C. 5 *μ*L of the reaction product were run in 1.5% (w/v) agarose gel stained with GelRed (Biotium, Hayward, CA, USA). Positive amplicons were cleaned using the UltraClean PCR Clean-up Kit (MO BIO, Carlsbad, CA, USA), cloned using a TOPO TA cloning kit (Life Technologies), and transformants were plated as described above. Positive, white colonies were picked and inoculated into 2 mL 2X Yeast extract/Tryptone and Ampicillin (0.05 mg mL^−1^) and grown overnight at 37°C with shaking at 50 rpm. Plasmids were purified using a FastPlasmid mini kit (5′) and sequenced at the Oregon National Primate Research Center with M13F (5′- TGT AAAACGACGGCCAGT-3′) and M13R (5′- AGGAAACAGCTATGACCAT-3′).

**Table 3:** Primers used for analysis of the unique sequence element (USE) detected in *Katablepharis* CRE.

Primer name	Sequence (5′→3′)	Target group	rRNA gene region	Research question	Reference
Kj3F	TGGATCGAAAGGTCTGGGTA	Katablepharidaceae	SSU	1	This study
Kj281R	TCCTCTGACTTCACCCTGCT	Katablepharidaceae	LSU	1	This study
528f	CCGCGGTATTCCAGCTC	Eukaryota	SSU	2	Elwood et al. (1985)
K28VR6	CCAACGGCAACAATTGACTA	*Katablepharis* CRE	LSU-USE	2, 3	This study
K28VF6	GGAATTAGGCCAGCATCAGA	*Katablepharis* CRE	LSU-USE	3	This study
ORF1	GACTCCTTGGTCCGTGTTTCAAGA	Eukaryota	LSU	2	This study
ORF2	CGAACAAGTACCGTGAGGGAAAGATGCAAA	Eukaryota	LSU	2	This study

Research question refers to the questions discussed in the introduction.

### Quantitative PCR analysis of estuarine samples for the *Katablepharis* CRE USE

Quantification of the *Katablepharis* CRE USE was performed for samples collected from March to July 2013. To do so, forward (K28VF6: 5′- GGAATTAGGCCAGCATCAGA-3′) and reverse (K28VR6: 5′- CCAACGGCAACAATTGACTA-3′) primers were designed to amplify a 144 bp sequence unique for the USE of *Katablepharis* CRE. Primer specificity was tested through end-point PCR followed by TOPO cloning and sequence analysis (described above), and PCR conditions were optimized to minimize primer-dimer formation. All sequences recovered from this PCR were highly related (>99%) to the USE of *Katablepharis* CRE. The qPCR reactions were performed in a final volume of 20 *μ*L containing 10 *μ*L Synergy Brands (SYBR) Green I PCR Master Mix (Life Technologies), 8 *μ*L water, 0.25 *μ*mol/L of each primer, and 1 *μ*L template. All reactions were performed on a StepOnePlus Real-Time PCR system (Life Technologies), with an initial denaturation step (94°C, 2 min) followed by forty cycles of 15 sec of denaturation at 94°C, annealing-extension at 60°C for 1 min, and 15 sec of data collection at 81°C. Melting curve analysis was performed to assess nonspecific amplification of primer-dimers, as SYBR green I binds to all double-stranded DNA without specificity. The dissociation curve from 60 to 95°C was measured after the last qPCR cycle and the melting temperature (*T*_m_) of potential primer-dimers and the specific PCR products was obtained. The majority of samples contained a single melting temperature peak with no evidence of primer-dimer formation; however, in order to suppress fluorescence caused by primer-dimer formation (*T*_m_ ∼65°C), the temperature of the detection step was set above that of primer-dimers but below that of the specific PCR product (∼85°C).

A plasmid-bearing cloned USE for *Katablepharis* CRE was constructed and linearized to use as standards for qPCR. Six standard reactions with concentrations ranging from 4.07 × 10^0^–4.07 × 10^5^ gene copies *μ*L^−1^ were used to construct standard curves. The concentration of genomic insert DNA from linearized plasmids was measured fluorometrically using a Qubit® 2.0 flourometer (Life Technologies). The number of gene copies in the standard was calculated as:
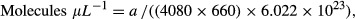
where *a* is the plasmid DNA concentration (g *μ*L^−1^), 4080 is the plasmid length, including the vector (3931 bp) and inserted PCR fragment (149 bp), 660 is the average molecular weight of one base pair, and 6.022 × 10^23^ is the number of molecules in a mole of a substance. Environmental samples, standards and blanks (water as template) were run in triplicate and 1 *μ*L DNA sample were used per reaction. The concentration of targeted LSU rDNA (copies mL^−1^) was calculated from the following formula:
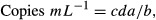
where *c* is the LSU rDNA concentration estimated by qPCR (copies *μ*L^−1^), *d* is the sample dilution factor, *a* is the volume of solution the DNA extract was resuspended (*μ*L), and *b* is the volume of water filtered (mL). qPCR data is available in Table S1.

### FISH analysis of estuarine samples for *Katablepharis* CRE

In order to estimate cell abundance and gain a sense of cell morphology of *Katablepharis* CRE, an oligonucleotide probe labeled with Alexa Fluor 555 (Life Technologies) specific for the USEof *Katablepharis* CRE was designed (VR113: 5′-GGAATTAGGCCAGCATCAGA-3′). FISH was conducted for a subset of samples from the 2013 time series that represented a mix of different depths, locations, and sampling dates (indicated by asterisks in Fig. 5B). For each sample, aliquots were fixed with paraformaldehyde (4% final concentration) and stored at 4°C. Protocols for detection of specific protist taxa by FISH have been reported previously (Pernthaler et al. 2001; Massana et al. 2006). Briefly, 10 mL fixed aliquots were filtered on 0.6 *μ*m pore size polycarbonate filters and hybridized for 3 h at 46°C in the appropriate buffer (with 30% formamide) (Pernthaler et al. 2001; Massana et al. 2006), washed at 48°C in a second buffer (Pernthaler et al. 2001; Massana et al. 2006), counter-stained with 4′,6-diamidino-2-phenylindole (DAPI; 5 *μ*g *μ*L^−1^) and proflavin (Sherr et al. 1993), and mounted in a slide. Cells were then observed and counted by epifluorescence microscopy (Axiovert 200M; Zeiss, Jena, Germany) on 400× magnification with oil immersion under a Cy3 filter. Cell concentration was calculated with the following formula:
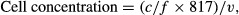
where *c* is the total number of cells counted, *f* is the total number of fields of view used, 817 is the number of field of views per filter on 400× magnification, and *v* is the volume filtered. A minimum of 20 fields of view were inspected.

## Results

### Heterotrophic protist assemblages in the Columbia River coastal margin

**Figure 3:**
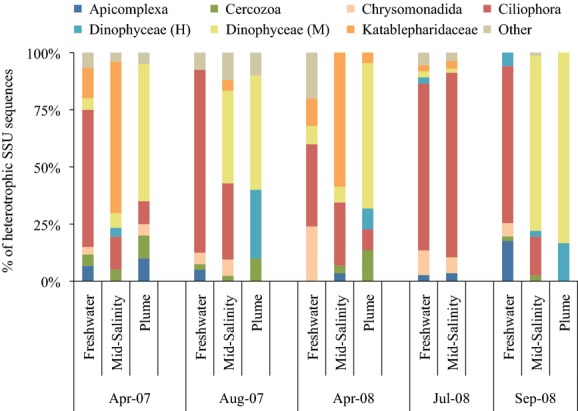
Percent composition of heterotrophic protists at the class level based on analysis of SSU sequence data for water collected in the Columbia River estuary and its plume in April and August 2007, and in April, July and September 2008. Freshwater = salinity of 0; Mid-Salinity = salinity of 15; Plume = salinity of 28–31. “H” refers to putative heterotrophic dinoflagellates, while “M” indicates putative mixotrophic dinoflagellates. “Other” category designates sequences associated with the following protist taxa: Bicosoecida, Centroheliozoa, Choanoflagellatea, Ichthyosporea, Labyrinthulida, Stramenopile MAST-12 group, Oomycetes, Pirsonia, and Telonemida. The dominance of Katablepharid sequences in April 2007 and 2008 mid-salinity waters denotes the genus *Katablepharis*.

Nucleotide sequence analysis of SSU rRNA gene DNA from the clone libraries was performed to examine seasonal and inter-annual variations of heterotrophic protist assemblages in the Columbia River estuary and its plume during the spring, summer, and autumn of 2007 and 2008. The most abundant heterotrophic protist sequences detected in the freshwater samples were attributable to ciliates (with 97–100% similarities at the genus level; Fig.3). Ciliate sequences represented a large proportion of all freshwater samples, with April samples consisting primarily of *Rimostrombidium*, which was also present in August 2007 along with the tintinnid genus *Tintinnopsis*. The genus *Stokesia* of the order Peniculida was particularly frequent in July and September 2008, while *Pelagodileptus* was also abundant in September 2008. Heterotrophic chrysomonad sequences were detected in all freshwater samples, with sequences related to *Paraphysomonas* accounting for high proportions of total sample clones in April 2008, while katablepharid sequences related to *Katablepharis japonica*, a small (∼5 *μ*m) heterotrophic flagellate, were detected in both April samples as well as August 2007.

In both April 2007 and 2008, mid-salinity (salinity ∼15) water samples were taken in the south channel of the estuary (Fig.1). SSU sequences were dominated by those belonging to the Katablepharidaceae, a class of colorless heterotrophic flagellate (Fig.3). The overwhelming majority of sequences relating to the class Katablepharidaceae in the April mid-salinity samples most closely resembled the SSU (18S) sequence of *Katablepharis japonica* (98% identity). These sequences also represented a large proportion of all sequences collected, including both heterotrophic and autotrophic protists in the April mid-salinity samples. Similar to the freshwater sample, ciliates were the dominant heterotrophs in the July 2008 mid-salinity water sequences. The majority of the retrieved ciliate sequences were related to *Stokesia*, which was found in comparable relative abundance (25% and 26%) in the July and September 2008 freshwater samples (Fig.3). In August 2007 and September 2008 mid-salinity samples, dinoflagellate sequences relating to the dinoflagellate *Gyrodinium*, a diverse genus that can be autotrophic, mixotrophic, or heterotrophic, were particularly frequent, ranging from 20% to 35% of heterotrophic sequences (Fig.3). *Gyrodinium* sequences were also the dominant dinoflagellate sequences in the plume samples from August 2007 and September 2008, while *Alexandrium*, *Pentapharsodinium*, and *Peridinium* represented most of the dinoflagellate sequences found in the other plume samples.

### LSU sequence analysis of *Katablepharis* CRE

**Figure 4:**
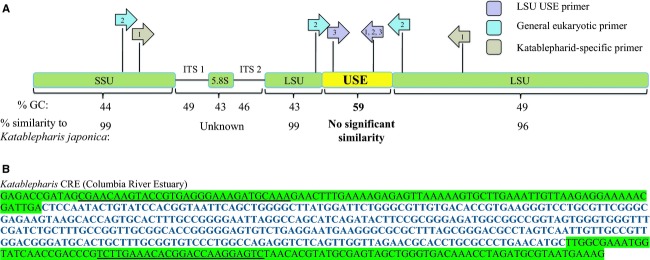
A. Sequencing of the rRNA gene of *Katablepharis* CRE revealed a 332 bp region of the 28S rRNA gene that is unique to the CRE strain. This region is GC-rich compared to the rest of the gene and shows no significant similarity to other katablepharids in the NCBI database, while the rest of rRNA gene aligns well with *K. japonica* and other sequenced katablepharids. Colored arrows indicate PCR primers designed in the 28S variable region (purple), to be general for all eukaryotes (teal), or katablepharid-specific (light brown). Numbers within the arrows refer to the primer sets that were used to answer the research questions discussed in the introduction. B. Nucleotide sequence of the *Katablepharis* CRE USE (bold) with flanking LSU sequence (green). ORF primers, general eukaryotic primers used to amplify additional D2 USE, are underlined.

To provide higher resolution of the genetic variability of the highly dominant katablepharid referred to herein as *Katablepharis* CRE, a full sequence of the ITS 1, 5.8S, and ITS 2 region and a partial sequence of the LSU of the rRNA gene were obtained from *Katablepharis* CRE (accession number KJ925151). The ITS1-5.8S-ITS2 region of *Katablepharis* CRE is a 667 bp sequence that is ∼93% similar to an ITS1-5.8S-ITS2 sequence of a cultured strain of *Katablepharis japonica* (CCMP 2791) from the Neuse River estuary in North Carolina (Fig.4). A 332 bp USE was uncovered in the *Katablepharis* CRE LSU gene at the 138th nucleotide from the 5′ end of the sequence. This region showed no significant similarity to any LSU rRNA sequence in the NCBI database (last checked 3 January 2014). However, the sequences down- and upstream of this USE region show 99% and 96% similarity to the *K. japonica* sequence in the NCBI database (accession number FJ973371), respectively. Another intriguing feature of the USE region is the difference in GC content of the variable region compared to its flanking regions. The USE has a GC content of 59%, while the down- and upstream regions are both <50%.

To assess the diversity and distribution of the USE in *Katablepharis* CRE, and potentially other organisms, a suite of PCR primers were used (Table3), and positive amplifications were cloned and sequenced. To determine how widespread the USE is amongst *Katablepharis* CRE and other katablepharids in the Columbia River coastal margin, PCR was performed using katablepharid-specific SSU forward Kj3F and LSU reverse (Kj281R: 5′-TCCTCTGACTTCACCCTGCT-3′, position:947–966) primers flanking the USE (Fig.4). All amplified products were cloned and sequenced, and all recovered sequences were related to one of two katablepharids: *Katablepharis* CRE or *Leucocryptos*. All sequences related to *Katablepharis* CRE recovered from the estuary contained the D2 region USE. The only other katablepharid detected in the system (*Leucocryptos*) did not have this element, but rather contained an LSU sequence that aligned well (>97% similarity) with the *K. japonica* (accession number FJ973371) and *L. marina* (accession number DQ980471) sequences in the NCBI database in this region.

To analyze the diversity and extent of USE in other organisms, or other systems outside the Columbia River coastal margin, a primer set consisting of a general eukaryotic forward primer (528f), and a USE-specific reverse primer (K28VR6) were employed to amplify sequences from all eukaryotic organisms that contained this region. Locations of water samples used for attempted amplification included the Columbia River coastal margin as well as Puget Sound, Grays Harbor Washington, Amazon River plume, Beaufort Sea, and several Russian rivers (see Crump et al. 2009 for sampling details). A total of 32 samples were analyzed but positive PCR amplification of the USE sequences occurred only in the Columbia River estuary samples, with all recovered sequences being identical to *Katablepharis* CRE. Several Columbia River estuary locations upriver of SATURN-04, in the freshwater tidal zone of the estuary, were also tested. However, all of these samples failed to amplify. This element also failed to align with the NCBI and Cyberinfrastructure for Advanced Microbial Ecology Research and Analysis (CAMERA) metagenomic databases. However, a similar sequence (96% identity) was detected in a metagenome collected from surface water off the Delaware coast during May 2010 (source IMG-MER).

To determine if other similar elements exist in other organisms, general forward (ORF1: 5′-GACTCCTTGGTCCGTGTTTCAAGA-3′) and reverse (ORF2: 5′-CGAACAAGTACCGTGAGGGAAAGATGCAAA-3′) primers that flank the USE were designed and used in PCR reactions (Fig.4). Resulting amplicons revealed unique elements in other protists at about the same location and of the same approximate size (294–400 bp). For example, a sequence related to the parasitic dinoflagellate *Eudubosquella* sp. Ex *Favella arcuata* (accession number JN934989) amplified from the Beaufort Sea lagoon near Alaska contained a 294 bp region. A sequence, related to *Eudubosquella* sp. Ex *Favella arcuate*, was also detected from the Columbia River coastal margin and contained a 292 bp region. The sequences flanking the elements align to each other, but the elements themselves do not align with each other or any other sequence in the NCBI database (analysis done on 3 January 2014).

### Distribution of *Katablepharis* CRE assessed through qPCR

**Figure 5:**
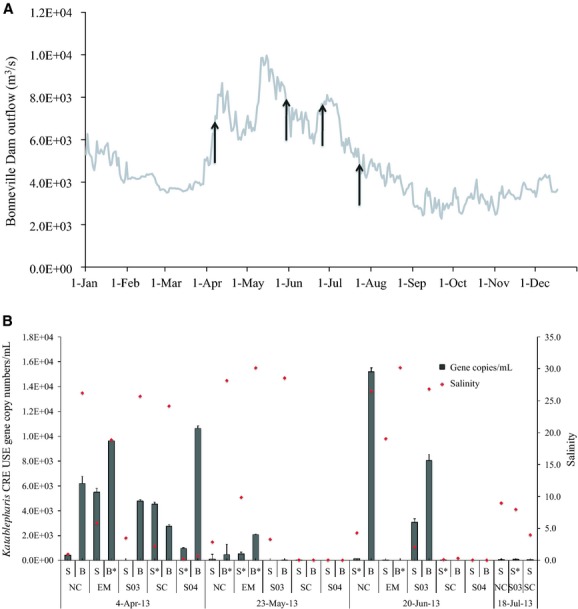
A. Annual Columbia River discharge (m3/s) measured at the outflow of Bonneville Dam for 2013 (daily mean). Gray arrows indicate sampling dates for qPCR and FISH analyses. B. Distribution of *Katablepharis* CRE USE in the Columbia River estuary estimated by qPCR from March–July 2013. NC refers to samples collected from the North Channel, EM in the estuary mouth, S03 near the SATURN-03 observatory station, SC in the South Channel, and S04 near the SATURN-04 observatory station (see Fig. 2 for exact locations). S = surface water; B = bottom water. Asterisks denote samples which were also analyzed with FISH. Error bars indicate standard deviation for each sample. Red dots indicate salinity.

While the use of SSU sequence libraries provides a snapshot of the protist assemblage and suggests that *Katablepharis* CRE is one of the dominant heterotrophic protists in the Columbia River estuary during spring, it is difficult to quantify protist populations using this method due to potential PCR biases and differences in gene copy numbers among protists (Heywood et al. 2011). To determine the distribution of *Katablepharis* CRE in the Columbia River coastal margin, USE-specific primers were designed and qPCR was performed for samples collected monthly between March and July 2013. The sampling period captured the annual spring freshet, with outflow from Bonneville Dam beginning to increase around the beginning of April (Fig.5A). During this period *Katablepharis* CRE was detected at all five sites within the estuary (salinities ranging from 0 to 26) with USE gene copy numbers as high as 1.06 × 10^4^ copies mL^−1^ measured (Fig.5B). These values are comparable to those found in samples from mid-salinity waters in April 2007 and 2008, where *Katablepharis* CRE USE gene copy numbers were present at 9.89 × 10^3^ and 1.09 × 10^4^ copies mL^−1^, respectively (data not shown). For the May 2013 samples, USE gene copy numbers were lower at each site than they were for the April 2013 samples, with the highest USE gene copy numbers measured at the bottom estuary mouth sample (8.1 m depth) with 2.09 × 10^3^ copies mL^−1^ (Fig.5B). However, USE gene copy numbers did increase again in three June samples, with the highest gene copy numbers of all samples (1.52 × 10^4^ copies mL^−1^) occurring in the bottom sample waters at the North Channel site characterized by a salinity of 26.5 and turbidity of 24.4 NTU, which was more than double the turbidity observed in the April or May samples at the same site and depth (9.5 and 11.1 NTU, respectively). There was an increase in river outflow as well during early June (Fig.5A), likely caused by rain events during late May and early June that brought 89.5 mm of precipitation to Astoria, Oregon between 24 May 2013 and 19 June 19 2013 (source: NOAA National Climatic Data Center). All copy numbers for the July samples were below 1.00 × 10^2^ copies mL^−1^. By this sampling date, river outflow had nearly returned to pre-freshet levels (Fig.5A).

### FISH of natural samples for *Katablepharis* CRE

**Figure 6:**
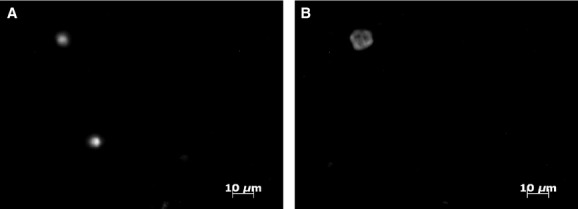
Epifluorescence micrographs of microorganisms larger than 0.6 μm (10 mL filtered) in Columbia River estuary water collected at the surface of the North Channel station on May 23, 2013. A. DAPI-stained cells and B. the corresponding microscopic field using the FISH *Katablepharis*-specific probe. Probe hybridized to a ∼7 m organism, the approximate size of katablepharids found in other systems.

**Figure 7:**
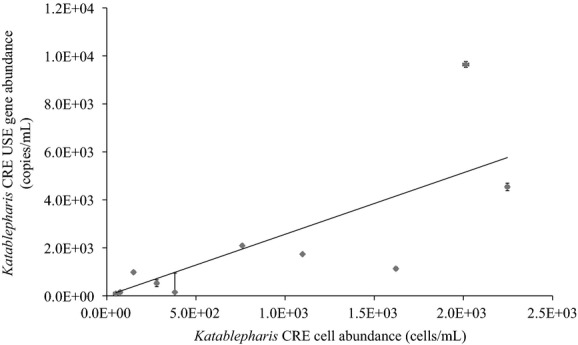
Comparison of *Katablepharis* CRE abundance estimated by FISH and qPCR using probes specific for its Unique Sequence Element (USE) within the large subunit for both approaches. The x axis corresponds to numbers of cells estimated through FISH and the y-axis to LSU copies measured by qPCR. Regression with y-intercept set to zero was used to estimate gene copy number per cell, with a slope of 2.6 and an R2 value of 0.59. Horizontal and vertical error bars indicate standard deviation for abundance estimates by FISH and qPCR, respectively.

Fluorescence in situ hybridization was conducted for a subset of samples (Table3 and samples marked by an asterisk on Fig.5B) in order to gain a sense of cell size and morphology and to estimate LSU gene copy number per cell of *Katablepharis* CRE. The fluorescent probe hybridized to ribosomal DNA within a ∼7 *μ*m organism (Fig.6), which is the approximate size of katablepharids in other systems (Okamoto and Inouye 2005). The abundance of *Katablepharis* CRE estimated by qPCR was plotted against that obtained by FISH counts, with the slope of the regression provided to estimate the gene copy number for *Katablepharis* CRE (Fig.7) (Zhu et al. 2005). There was a weak positive correlation between qPCR and FISH estimates of abundance, and the slope of the regression suggests a ratio of 2.5 gene copies per cell. Similarto other comparisons of FISH and qPCR using environmental samples (Rodríguez-Martínez et al. 2009), the *R*^2^ value (0.58) for this correlation is low, likely because biases in DNA extraction and qPCR optimization, as well as loss of cell integrity from fixation, may cause discrepancies between the two methods (Zhu et al. 2005).

## Discussion

A major goal of this study was to offer new insight into the heterotrophic protist assemblages of the Columbia River coastal margin through the use of molecular methods. The most notable, and unexpected, finding from our initial survey study was the dominance of SSU sequences resembling that of the heterotrophic flagellate class Katablepharidaceae in the mid-salinity (∼salinity = 15) samples in both April 2007 and 2008. Katablepharid SSU sequences comprised >40% of all the SSU sequences observed in each of these two samples. No other individual sequence, heterotrophic or autotrophic, during the 2-year time series was found in such high relative proportions. While it is not uncommon for autotrophic taxa to reoccur in high abundance, such as annually recurring spring blooms (Sverdrup 1953), this same phenomenon is less commonly observed in heterotrophic taxa.

In our study, *Katablepharis* sequences were most prominent in the estuarine clone libraries in April, with only a few sequences retrieved from the Columbia River plume libraries (Fig.3). Abundance estimates of these plume samples determined through qPCR using the *Katablepharis* CRE USE-specific primers were also relatively low compared to those in the estuary, with only 0.3 and 34.6 gene copies mL^−1^ observed in April 2007 and 2008, respectively (data not shown). *Katablepharis* has been found to be an important primary consumer and bacterivore associated with particles in freshwater and estuarine environments (Ploug et al. 2002; Domaizon et al. 2003; Slapeta et al. 2006). Events occurring in the spring, such as upriver diatom blooms and spring runoff, deliver organic matter to the estuary (Sullivan et al. 2001) that could fuel katablepharid proliferation. Indeed, as freshwater phytoplankton enter more saline waters they tend to lyse due to osmotic stress, releasing organic matter (Frey et al. 1984). *Katablepharis* CRE is likely an essential and yet previously undetected link between the microbial and herbivorous food webs in the Columbia River estuary. The USE marker identified herein provides a tool with which to investigate temporal and spatial dynamics of this organism that can be used to investigate potential trophic linkages.

Grazing by heterotrophic protists such as *Katablepharis* CRE can be an important force in shaping bacterial populations, transferring prey carbon to higher trophic levels, and remineralization of nutrients such as nitrogen and phosphorus (Caron et al. 1990). This information is particularly important in the Columbia River estuary, as it is often classified as a detritus-driven system, fueled by allochthonous organic matter with high levels of bacterial growth and productivity (Crump and Baross 1996). Through their role as bacterivorous grazers, *Katablepharis* and other heterotrophic protists provide a critical link between bacterial production and higher trophic levels, including invertebrates and fish. The identification of *Katablepharis* as an abundant taxon of heterotrophic protist within the SSU rRNA gene sequence data in the estuary during April 2007 and 2008 exemplifies the strength of molecular tools in illuminating taxonomic diversity in microbial assemblages, as this flagellate is a small, nonpigmented, nondescript eukaryote that could easily go unidentified using microscopic cell counting methods and undetected through pigment analyses.

The discovery of the USE within the LSU of *Katablepharis* CRE provided an excellent reagent to track the distribution of *Katablepharis* CRE in the system through USE-specific probes combined with quantitative and qualitative methods. Unlike ITS, another commonly used discriminating genetic marker, the USE sequence is an integral part of the LSU rRNA and is not removed by pre-rRNA processing. Hence, it presents a high copy target for probing abundance and distribution of protist variants. This yielded a specific qPCR assay that provided quantitative data and allowed for visualization of this uncultured katablepharid through FISH. This qPCR assay confirmed the wide distribution of *Katablepharis* CRE in the Columbia River estuary with respect to salinity (0–26), but with overall higher abundances measured in bottom waters at each site. Salinity is often a key determinant of protist populations as halotolerance differs among species (Frey et al. 1984), but other heterotrophic flagellates, such as marine strains of *Bodo designis*, can survive and grow at a wide range of salinities (Koch and Ekelund 2005). Molecular diversity studies of heterotrophic flagellate communities from other brackish systems such as the Baltic Sea also indicate a dominance of putative marine species that can survive brackish to fully marine salinity ranges (Weber et al. 2012). While *Katablepharis* CRE was detected at a wide range of salinities, its geographic range in the estuary seems to be constrained to the portion of the estuary affected by salinity intrusion. To verify this, additional freshwater samples collected in April 2013 as far upriver as the SATURN-05 observatory station near Longview, Washington were also assayed by qPCR using the *Katablepharis* CRE USE-specific primers, but *Katablepharis* CRE was not detected any further upriver than SATURN-04 (data not shown), which is located near the limit of salinity intrusion during April (Fig.1).

The presence of the USE in *Katablepharis* CRE and other protists has several potential important evolutionary and ecological implications. It is found within the D2 region of the LSU, which can contain variable length and be more divergent than the rest of the LSU gene (Hassouna et al. 1984). However, the unique element of *Katablepharis* CRE confers extreme variability compared to the rest of the D2 region, with no significant similarity to any sequences in the NCBI database. It also contains elevated GC content compared to the rest of the associated rRNA genes, possibly as a result of lateral gene transfer, a process that may be more prevalent in phagotrophic protists than previously thought (Andersson 2005). The USE sequences that have been uncovered can greatly increase taxonomic resolution of LSU protist diversity, be utilized for qualitative and quantitative monitoring through strain-specific probes, and increase the taxonomic resolution of ecogenomic technologies such as the environmental sample processor (Scholin 2010).

## Supporting Information

Figure S1 Percent composition of heterotrophic protists at the class level based on analysis of 500 bp SSU sequence data for water collected in the Columbia River estuary and its plume in April and August 2007, and in April, July and September 2008. Freshwater = salinity of 0; Mid-Salinity = salinity of 15; Plume = salinity of 28-31. “H” refers to putative heterotrophic dinoflagellates, while “M” indicates putative mixotrophic dinoflagellates. “Other” category designates sequences associated with the following protist taxa: Bicosoecida, Centroheliozoa, Choanoflagellatea, Ichthyosporea, Labyrinthulida, Stramenopile MAST-12 group, Oomycetes, Pirsonia, and Telonemida. The dominance of Katablepharid sequences in April 2007 and 2008 mid-salinity waters denotes the genus *Katablepharis*.

Table S1 Abundance of *Katablepharis* CRE USE in the Columbia River estuary estimated by qPCR from March to July 2013
