# Supplementary figures and images for: Discovery of a Katablepharis sp. in the Columbia River estuary that is abundant during the spring and bears a unique large ribosomal subunit sequence element
Source: Microbiologyopen. 2014 Aug 28;3(5):764–76. doi: 10.1002/mbo3.206 (PMC4234266; doi:10.1002/mbo3.206)

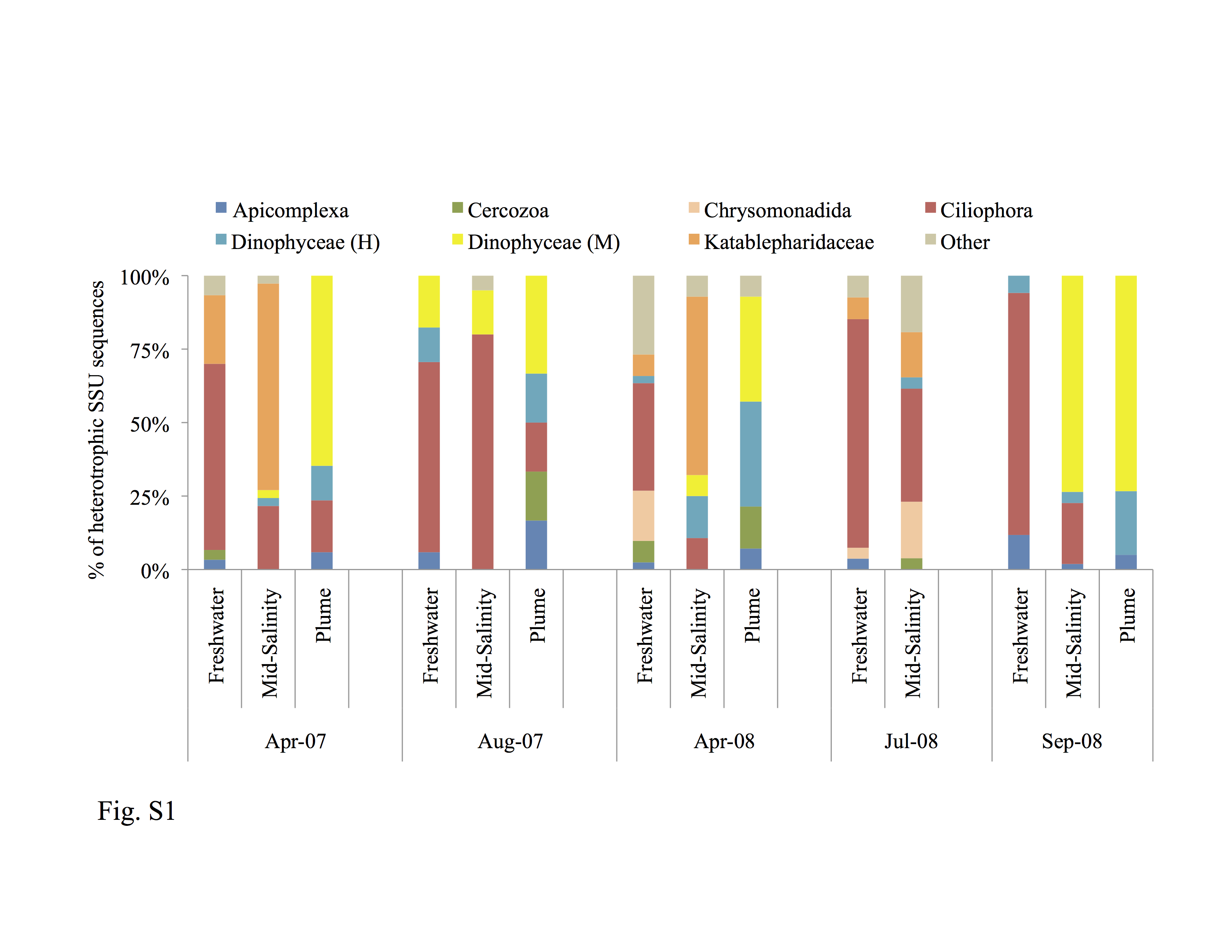

Supplement: Figure S1 — Percent composition of heterotrophic protists at the class level based on analysis of 500 bp SSU sequence data for water collected in the Columbia River estuary and its plume in April and August 2007, and in April, July and September 2008. Freshwater = salinity of 0; Mid-Salinity = salinity of 15; Plume = salinity of 28-31. “H” refers to putative heterotrophic dinoflagellates, while “M” indicates putative mixotrophic dinoflagellates. “Other” category designates sequences associated with the following protist taxa: Bicosoecida, Centroheliozoa, Choanoflagellatea, Ichthyosporea, Labyrinthulida, Stramenopile MAST-12 group, Oomycetes, Pirsonia, and Telonemida. The dominance of Katablepharid sequences in April 2007 and 2008 mid-salinity waters denotes the genus Katablepharis. [file mbo30003-0764-sd1.tiff]
