# Supplementary material for: Discovery of a Katablepharis sp. in the Columbia River estuary that is abundant during the spring and bears a unique large ribosomal subunit sequence element
Source: Microbiologyopen. 2014 Aug 28;3(5):764–76. doi: 10.1002/mbo3.206 (PMC4234266; doi:10.1002/mbo3.206)
Supplement: Table S1 — Abundance of Katablepharis CRE USE in the Columbia River estuary estimated by qPCR from March to July 2013 [file mbo30003-0764-sd2.docx]

| Date | Location | Depth | *Katablepharis* CRE gene copies mL ^-1^ | S.D. |
| --- | --- | --- | --- | --- |
| 4-Apr-13 | NC | S | 4.2E+02 | 1.2E+02 |
|  |  | B | 6.2E+03 | 5.8E+02 |
|  | EM | S | 5.5E+03 | 3.2E+02 |
|  |  | B | 9.6E+03 | 1.2E+02 |
|  | S03 | S | 5.8E+00 | 5.3E-01 |
|  |  | B | 4.8E+03 | 1.2E+02 |
|  | SC | S | 4.5E+03 | 1.6E+02 |
|  |  | B | 2.7E+03 | 1.3E+02 |
|  | S04 | S | 9.8E+02 | 4.1E+01 |
|  |  | B | 1.1E+04 | 2.2E+02 |
| 23-May-13 | NC | S | 1.1E+02 | 3.8E+02 |
|  |  | B | 4.8E+02 | 8.0E+02 |
|  | EM | S | 5.3E+02 | 1.4E+02 |
|  |  | B | 2.1E+03 | 3.0E+00 |
|  | S03 | S | 0.0E+00 | 0.0E+00 |
|  |  | B | 2.5E+01 | 3.0E+00 |
|  | SC | S | 3.8E+01 | 4.3E+01 |
|  |  | B | 0.0E+00 | 0.0E+00 |
|  | S04 | S | 0.0E+00 | 0.0E+00 |
|  |  | B | 0.0E+00 | 0.0E+00 |
| 20-Jun-13 | NC | S | 1.5E+02 | 1.9E+00 |
|  |  | B | 1.5E+04 | 3.3E+02 |
|  | EM | S | 2.7E+01 | 4.2E-01 |
|  |  | B | 2.6E-01 | 9.5E-03 |
|  | S03 | S | 3.1E+03 | 2.8E+02 |
|  |  | B | 8.0E+03 | 4.9E+02 |
|  | SC | S | 2.7E+01 | 2.4E+01 |
|  |  | B | 2.6E-01 | 7.1E+00 |
|  | S04 | S | 1.2E+00 | 8.4E-02 |
|  |  | B | 5.7E-01 | 8.2E-04 |
| 18-Jul-13 | NC | S | 8.2E+01 | 8.6E+00 |
|  | S03 | S | 8.9E+01 | 7.2E+00 |
|  | SC | S | 8.0E+01 | 2.3E+00 |

Table S1. Abundance of *Katablepharis* CRE USE in the Columbia River estuary estimated by qPCR from March-July 2013. NC refers to samples collected from the North Channel, EM in the estuary mouth, S03 near the SATURN-03 observatory station, SC in the South Channel, and S04 near the SATURN-04 observatory station (see Fig. 2 for exact locations). S = surface water; B = bottom water. S.D = standard deviation.
